# Ultrasound does not activate but can inhibit in vivo mammalian nerves across a wide range of parameters

**DOI:** 10.1038/s41598-022-05226-7

**Published:** 2022-02-09

**Authors:** Hongsun Guo, Sarah J. Offutt, Mark Hamilton II, Yohan Kim, Cory D. Gloeckner, Daniel P. Zachs, Jamu K. Alford, Hubert H. Lim

**Affiliations:** 1 Department of Biomedical Engineering, University of Minnesota, Minneapolis, MN 55455 USA; 2 Restorative Therapies Group, Medtronic, Minneapolis, MN 55432 USA; 3 Institute for Translational Neuroscience, University of Minnesota, Minneapolis, MN 55455 USA; 4 Department of Otolaryngology, Head and Neck Surgery, University of Minnesota, Minneapolis, MN 55455 USA

**Keywords:** Biomedical engineering, Peripheral nervous system

## Abstract

Ultrasound (US) has been shown to stimulate brain circuits, however, the ability to excite peripheral nerves with US remains controversial. To the best of our knowledge, there is still no in vivo neural recording study that has applied US stimulation to a nerve isolated from surrounding tissue to confirm direct activation effects. Here, we show that US cannot excite an isolated mammalian sciatic nerve in an in vivo preparation, even at high pressures (relative to levels recommended in the FDA guidance for diagnostic ultrasound) and for a wide range of parameters, including different pulse patterns and center frequencies. US can, however, reliably inhibit nerve activity whereby greater suppression is correlated with increases in nerve temperature. By prohibiting the nerve temperature from increasing during US application, we did not observe suppressive effects. Overall, these findings demonstrate that US can reliably inhibit nerve activity through a thermal mechanism that has potential for various health disorders, though future studies are needed to evaluate the long-term safety of therapeutic ultrasound applications.

## Introduction

Ultrasound (US) is emerging as a promising modality to noninvasively stimulate brain circuits, as well as skin and deep receptor structures in animals and humans^1–7^, in which single element or phased array transducers can be designed to focus energy into different targets within the body^8,9^. However, direct excitation of peripheral nerves with US remains controversial with inconsistent findings across studies^10–12^. Previous in vivo noninvasive studies have reported that US can excite nerves through intact skin and muscle, particularly the rat abducens nerve or mouse sciatic nerve with low stimulus intensities (relative to levels recommended in the FDA guidance for diagnostic ultrasound^13^; e.g., pressure of 0.53 or 2.6 MPa, respectively^11,12,14^). Other noninvasive US stimulation studies that recorded muscle action potentials as a surrogate for mouse sciatic nerve activity required much higher pressures, such as 13 or 28 MPa, for presumably exciting the nerve. Note that these pressures are much higher than the levels recommended in the FDA guidance for diagnostic ultrasound (e.g., intensity of 5633 W/cm^2^ that exceeds 190 W/cm^2^, and mechanical index of 6.5 that exceeds 1.9^13–16^). In contrast, when directly recording from the nerve, one study showed that US cannot elicit compound action potentials (CAPs) in mammalian nerves^17^, which is supported by a more recent study demonstrating that US cannot excite an ex vivo mouse sciatic nerve^18^. Considering the immense potential for using US as a noninvasive method to excite or modulate nerves in the body for a broad range of health conditions, including pain management, inflammation disorders, motor rehabilitation, autonomic dysfunction, and spasticity^19–23^, further studies are critically needed for resolving those contradictory US results and for identifying effective parameters for nerve stimulation.

There is greater evidence of the ability of US to inhibit peripheral nerve activity rather than elicit direct excitation, including studies published several decades ago showing that US can reversibly or irreversibly block mammalian peripheral nerves^17,24,25^. Recent studies have also demonstrated that US can modulate nerve activity in both in vitro and in vivo animal preparations^26–32^. However, in mammalian experiments, US was either transcutaneously applied to the nerves^26,31,32^ or was applied to nerves not being fully isolated from surrounding tissue^17,30^, which may have confounding effects of stimulation of muscle and skin. Additionally, there are discrepancies on the types of nerve modulation achievable with US stimulation (i.e., enhanced versus suppressed activity)^10^ and the effective range of US parameters, such as related to duty cycle, stimulation time, intensity, and center frequency of the US transducer. While thermal and non-thermal effects have both been proposed as mechanisms for US modulation of nerve activity^17,18^, there remains a lack of in vivo studies with control experiments to elucidate the mechanism of modulation and its operating range of US parameters.

In this study, we performed in vivo experiments in a guinea pig model to investigate if low-intensity US could directly excite or inhibit a well isolated mammalian sciatic nerve, and to evaluate the largest set of parameters that has been examined for US stimulation of peripheral nerves. In this study, we applied pressures below and above levels that have been recommended in the FDA guidance for diagnostic ultrasound. These FDA guidance levels are used as a reference point in our study. However, future studies will be required to confirm the safety of identified effective parameters when used for therapeutic applications rather than diagnostic purposes (e.g., higher intensities or lower intensities with longer periods of stimulation that are not covered in that FDA guidance, including the need for proper thermal safety calculations^13,33,34^). For our study, we applied US to the intact leg surface to noninvasively target the sciatic nerve, to the exposed sciatic nerve lying within the muscle cavity, and to the isolated sciatic nerve separated from the surrounding tissue. From these unique setups, we could identify different contributions of US-evoked activity (i.e., from stimulation of surrounding tissue versus the sciatic nerve) by recording the corresponding neural activity directly from the sciatic nerve and from the somatosensory cortex (SSC). To explore the mechanism of US stimulation of nerves, we also performed thermal control experiments in which the nerve temperature was regulated during US application. Overall, our results reveal that: (1) US activates the skin, surrounding tissue and/or muscles but cannot directly excite the myelinated sciatic nerve with numerous parameters tested using different US transducers and center frequencies; (2) US reliably inhibits nerve activity but direct enhancement of nerve activity is not possible; and (3) greater suppression of nerve activity is accompanied by larger increases in nerve temperature.

## Results

### Noninvasive ultrasound stimulation of sciatic nerve

**Figure 1:**
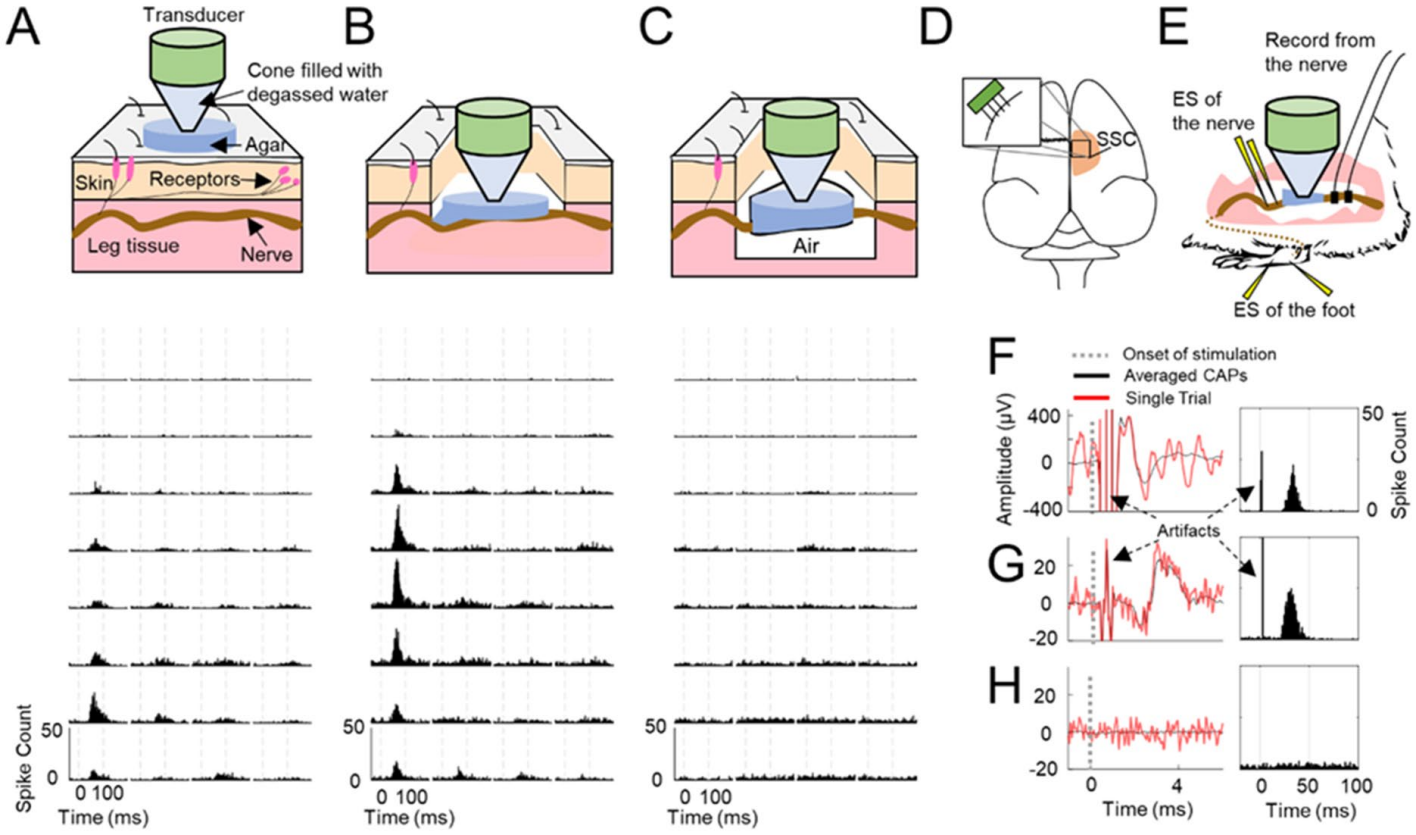
Experimental setup with example neural responses. **(A)** Noninvasive stimulation with skin, muscle, and nerve intact. The transducer was positioned above the skin overlying the sciatic nerve and coupled with agar. **(B)** Invasive stimulation with the transducer positioned in the muscle cavity over the exposed sciatic nerve and coupled to the nerve with agar. **(C)** Invasive stimulation with the sciatic nerve “hammocked” by the transducer with parafilm to isolate the nerve from the surrounding tissue to obtain nerve recordings with minimal contamination from non-nerve activity and to maximize the amount of ultrasound (US) energy that reaches the nerve versus the surrounding tissue/muscle (i.e., US waves do not readily travel through air). Somatosensory cortex (SSC) responses for A to C were recorded using the setup shown in D with a 32-site electrode array (4 shanks, 8 sites per shank) inserted into the SSC and in response to US stimulation (1 MPa, 200 Hz pulse repetition frequency [PRF], 2.5 ms pulse duration [PD], 10 pulses, 1 s trial duration [TD], 0.22 MHz center frequency), which are plotted as post-stimulus time histograms (PSTHs, 1-ms bins) across 100 trials (lower plots in A to C; 32 PSTHs shown for each setup). In A, US focus was at the surface of the intact skin, which is about 4 mm above the nerve. Referring to the pressure profile in Supplementary Fig. S1, the pressure at the nerve in A was approximately 60%, 80%, and 55% of that at the focus, for 0.22, 0.52, and 1 MHz transducer, respectively. In B and C, US focus was at the nerve, which is 1 mm below the plastic cone of the transducer. In (**C**), the total agar thickness is approximately 2.5 mm. **(D)** Diagram of preparation for recording multi-unit activity from SSC with a 32-site electrode array. **(E)** Diagram of preparation for recording compound action potentials (CAPs) in response to electrical stimulation (ES) of the foot, ES of the nerve, and US stimulation of the nerve. (**F–H)** Examples of the averaged CAPs in response to 100 trials of biphasic ES (205 µs/phase, 0.1 mA) of the sciatic nerve with platinum-iridium wire electrodes (**F**), biphasic ES (205 µs/phase, 2.82 mA) of the foot with stainless steel needle electrodes (**G**), and US stimulation (1.3 MPa, single pulse, 1 ms PD, 0.5 s TD, 0.52 MHz center frequency) of the sciatic nerve (**H**). Single trial CAPs were also plotted as red curves. Representative examples of PSTHs are shown in the right column. The onset time of ES or US stimulation was at zero (black dotted lines in **F** to **H**). Electrical artifacts are observed in the CAPs (black arrows in **F** and **G**).

Based on previous findings that US can noninvasively excite the mouse sciatic nerve and rat abducens nerve^11,12^, we designed a setup for performing noninvasive US stimulation of the guinea pig sciatic nerve (Fig. 1A) to assess if US can excite peripheral nerves. SSC activity was recorded in response to pulsed US with 0.22, 0.52, and 1 MHz transducers (Supplementary Fig. S1). US with different center frequencies and varying pulse repetition frequencies (PRFs) were all able to elicit SSC activity (Fig. 1A and Supplementary Fig. S2). A variety of other parameters (Supplementary Table S1), ranging from 0.4 to 5 MPa for pressure, 0.2 to 5 kHz for PRF, 0.1 to 50 ms for pulse duration (PD), 6 to 100 ms for stimulation on time per trial, and 0.5 s for trial duration (TD), as well as single pulses, showed the ability to elicit SSC activity across different animals. Parameters below and above the levels recommended in the FDA guidance for diagnostic ultrasound were able to elicit SSC activity (Supplementary Table S1).

### Nerve excitation is not possible with low-intensity ultrasound

Previous studies have shown that US activates receptors when applied directly on the skin^35,36^. Therefore, one major concern with our noninvasive preparation (Fig. 1A) is if the SSC activity was elicited in response to US activation of the surrounding skin and tissue in the leg instead of direct excitation of the sciatic nerve^37^. Additionally, in the noninvasive preparation, the nerve was about 4 mm below the focus of the transducer and therefore only received part of the US energy (55–80% for different transducers as explained in the caption of Fig. 1). To address these issues, we developed two additional experimental setups. In the first setup, the sciatic nerve was exposed by removing the overlying skin and muscle (Fig. 1B). In the second setup, the sciatic nerve was “hammocked”, thereby isolating the nerve from the surrounding tissue (Fig. 1C). In both setups, the nerve was positioned at the focus of the US transducer (i.e., 1 mm below the plastic cone tip, see Fig. S1). We tested several different center frequency transducers (0.22, 0.52, and 1 MHz) that are widely used in US neuromodulation studies^38^.

As shown in Fig. 1B, US stimulation of the exposed sciatic nerve, with the nerve still contacting the surrounding tissue and muscles, was able to elicit SSC activity similar to the responses observed in the noninvasive US preparation (Fig. 1A and Supplementary Fig. S2). Unexpectedly, when directly recording from the nerve, we did not observe any CAPs (Supplementary Fig. S3).

**Figure 2:**
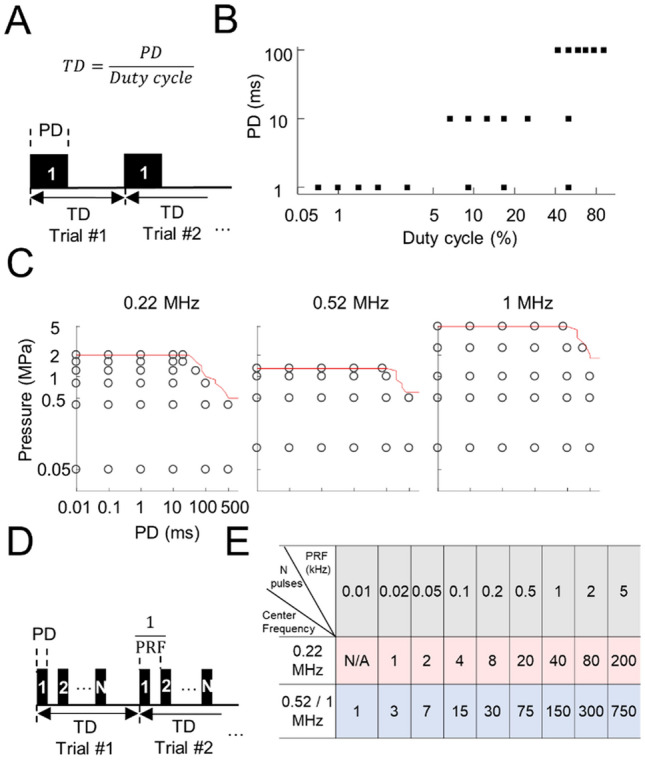
US does not excite the sciatic nerve using numerous parameters. **(A)** Single pulse stimulation patterns were used for the US experiments, where the duty cycle was defined as the PD divided by the TD and stimulation on time was defined as PD. **(B)** Single pulse paradigms, in which PDs at 1, 10, and 100 ms were tested with a variety of duty cycles and TDs. **(C)** Single pulse paradigms, in which PDs were exponentially chosen from 10 µs to 500 ms for 1 s TDs. The red lines indicate the maximum pressures usable to minimize potential damage to the transducers. The longest PDs tested at the highest pressures of US were 20, 75, and 75 ms for 0.22, 0.52, and 1 MHz, respectively. Different TDs of 0.5 and 3 s were also tested, in which the PDs were halved for 0.5 s TD. (**D)** Multiple pulse stimulation patterns were used for the US experiments, where duty cycle was defined as PD over the reciprocal of PRF and stimulation on time (per trial) was defined as PD times number of pulses per trial (N). (**E)** Multiple pulse paradigms, in which PRFs at 0.01, 0.02, 0.05, 0.1, 0.2, 0.5, 1, 2, and 5 kHz were tested with 50% duty cycle when US was presented for 20, 75, and 75 ms per trial (1 s TD) at the highest pressures of 2, 1.3, and 5 MPa for 0.22, 0.52, and 1 MHz transducer, respectively. None of these displayed paradigms elicited a CAP or any activity in SC, indicating that direct excitation of the sciatic nerve with US is not readily possible. These parameters (**B**, **C**, and **E**) were tested across ten animals. The maximum intensities tested for US stimulation substantially exceeded levels recommended in the FDA guidance for diagnostic ultrasound (*I*_SPTA_ of 62.48 W/cm^2^, *I*_SPPA_ up to 833 W/cm^2^, and mechanical index of 5 exceeding limits of 0.72 W/cm^2^, 190 W/cm^2^, and 1.9, respectively), yet still did not excite the nerve. Further description and equations for these intensity parameters are provided in Materials and Methods.

Additionally, when the nerve was isolated in the hammocked setup, US did not evoke neural activity in SSC (Fig. 1C) across a wide range of US parameters, even with higher intensity stimuli substantially exceeding (Fig. 2). These results suggest that low-intensity US is not able to directly excite the sciatic nerve, at least for the wide range of parameters tested in our study, and that SSC responses (Fig. 1A, B) were caused by US activation of receptors in the leg skin and/or surrounding tissue.

A potential limitation of recording neural activity in SSC is that our electrode sites may not be sensing neural responses projecting from the sciatic nerve. To address this issue, we recorded directly from the sciatic nerve using our hammocked setup during US stimulation. To rule out that the hammocked preparation damaged the sciatic nerve, limiting its ability to be activated by US, we periodically (in about every 30 min) stimulated the sciatic nerve with 100 trials of electrical stimulation (ES) to the nerve itself or to the foot to confirm that responses could still be sensed by our nerve recording electrodes (Fig. 1E). ES of the sciatic nerve and of the foot elicited large CAPs recorded from the nerve, as well as strong SSC responses (Fig. 1F, G). However, US stimulation of the nerve did not elicit any noticeable activity from the nerve or SSC (Fig. 1H). The success of eliciting nerve and SSC activity electrically confirmed that the nerve was still functional; thus, failure to evoke nerve or SSC activity with US was not due to a damaged sciatic nerve. By testing numerous US parameters depicted in Fig. 2, we verified that low-intensity US was not able to directly excite the sciatic nerve for a wide range of parameters, including the use of continuous mode (Fig. 2A–C) or pulsed mode with varying pressures, duty cycles, PDs, and PRFs (Fig. 2D, E). The inability to excite the sciatic nerve with US was true whether the nerve was hammocked or the nerve was immersed within the muscle cavity without the hammocked setup (Supplementary Fig. S3), where the agar-air boundary was removed to minimize potential confounds caused by standing waves at the location of the nerve.

### Ultrasound suppresses nerve activity

Although nerve excitation was not possible with the wide range of US parameters, we still investigated whether US could directly modulate nerve activity. Modulatory effects were characterized by electrically stimulating the sciatic nerve and measuring the change in neural activity caused by US stimulation of the nerve. To minimize confounding activation effects caused by US stimulation of surrounding tissue and skin receptors (Fig. 1A, B, and Supplementary Fig. S3), we used our hammocked nerve preparation (Fig. 1C, E). Since electrical stimulation of the nerve can unintentionally activate surrounding tissue and muscle, which can confound or mask the direct recordings from the nerve^39^, we instead applied ES to the foot. This preparation enabled sufficient activation of the sciatic nerve without any noticeable muscle responses and with smaller electrical artifacts (further described in Materials and methods).

**Figure 3:**
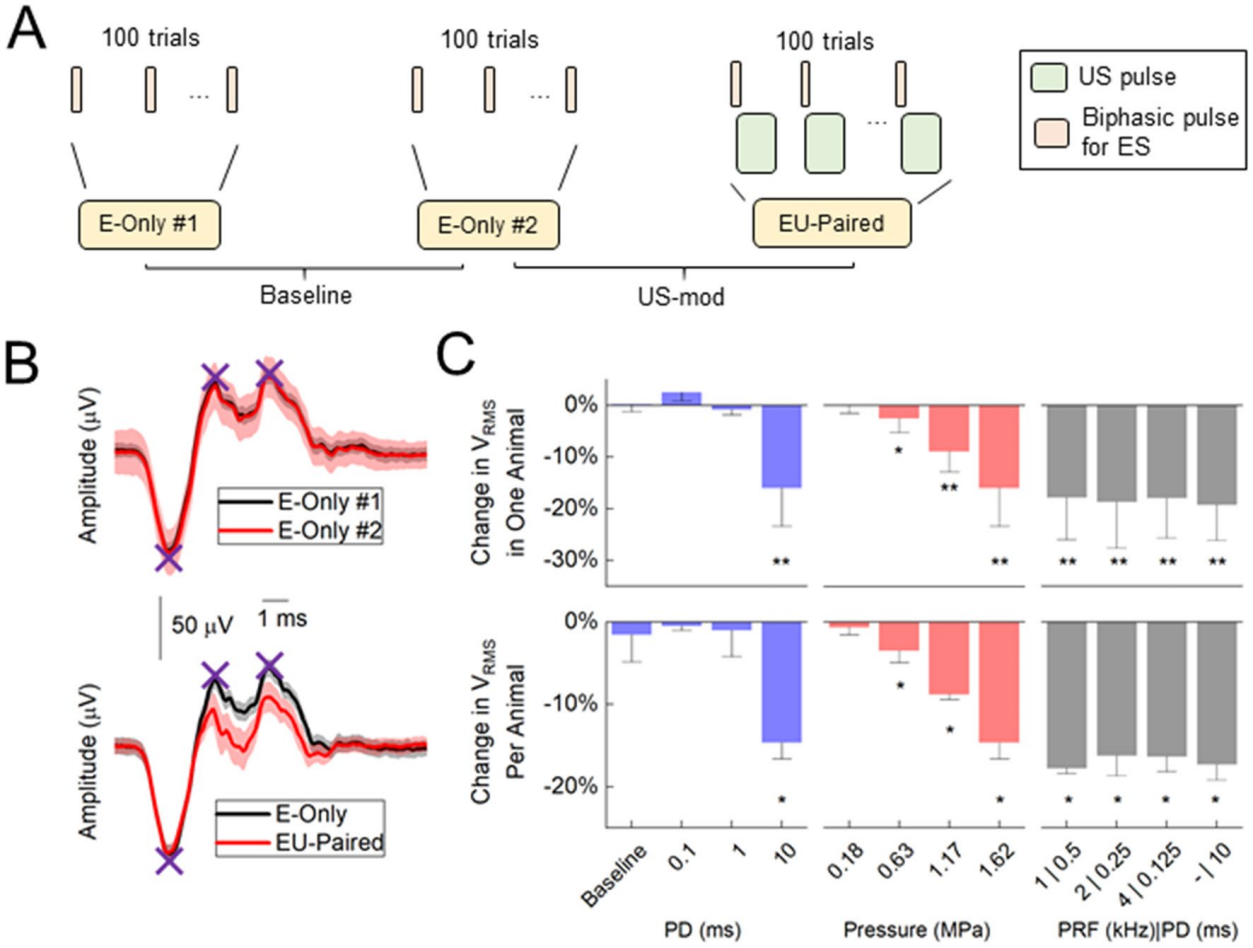
US modulation of the sciatic nerve depends on PD and pressure. **(A)** Diagrams of baseline and US modulation (US-mod) paradigms. Baseline paradigm compares the *V*_RMS_ between two consecutive E-Only sessions, while US-mod paradigm compares *V*_RMS_ between an E-Only session (nerve response to ES of foot stimulation) and an EU-Paired session (US stimulation of nerve that has been activated from ES of foot). **(B)** Examples of changes in CAPs for baseline and US-mod paradigms. The US parameters used in the EU-Paired were 1.62 MPa, 10 ms PD, 1 s TD, and 0.22 MHz center frequency. Standard deviation (SD) of CAPs across the 100 trials are plotted as black and red shadows. Purple Xs mark negative and positive peaks used for calculating *V*_RMS_ (described in Materials and Methods). **(C)** A set of US parameters were tested. Changes in *V*_RMS_ (mean ± SD) across 100 trials in one animal (upper) and across animals (n = 3; lower) are plotted. PDs varied from 0.1 to 10 ms (1.62 MPa), pressures varied from 0.18 to 1.62 MPa (10 ms PD), and PRFs varied from single pulse to multiple pulses with 1 to 4 kHz (10 ms stimulation on time per trial, 1.62 MPa). * indicates nerve activity in the US-mod paradigm was significantly suppressed (*p* < 0.05) compared with that in baseline paradigm. ** indicates *p* < 0.0045 to account for multiple comparisons based on Bonferroni correction (11 comparisons per plot). The 0.22 MHz transducer was used. Data are represented as mean ± SD.

Modulatory effects were calculated by taking the differences between the *V*_RMS_ of CAPs elicited by ES of the foot alone (E-only) and the *V*_RMS_ of CAPs during paired ES of the foot and US stimulation of the ipsilateral sciatic nerve (EU-paired; i.e., to assess how US modulates the ongoing nerve activity caused by E-only). Figure 3B presents an example showing that the CAPs were not significantly different between two consecutive E-only sessions (baseline paradigm in Fig. 3A; p > 0.05, statistical tests defined in Materials and methods). In contrast, the CAP example for EU-Paired (1.62 MPa, 10 ms PD, 1 s TD, 0.22 MHz) was significantly reduced by 17.35% compared with that of the preceding E-only (US-mod paradigm in Fig. 3A; p = 7.5E-7).

We further investigated how different PDs, pressures, and PRFs, in addition to a 10-ms single pulse, would affect US modulation of nerve activity with the 0.22 MHz transducer. One parameter was modified at a time, while keeping other parameters unchanged (Fig. 3C). We observed significant nerve suppression for longer durations (10 ms) or higher pressures (0.63, 1.17, and 1.62 MPa) of US stimulation. All tested PRFs showed significant suppression compared with baseline. There was no significant difference among PRFs (p > 0.05), suggesting that PRFs may not play a role if the stimulation on-time per trial (defined in Fig. 2A, D) and pressure are fixed, at least for the values used in our experiments.

**Figure 4:**
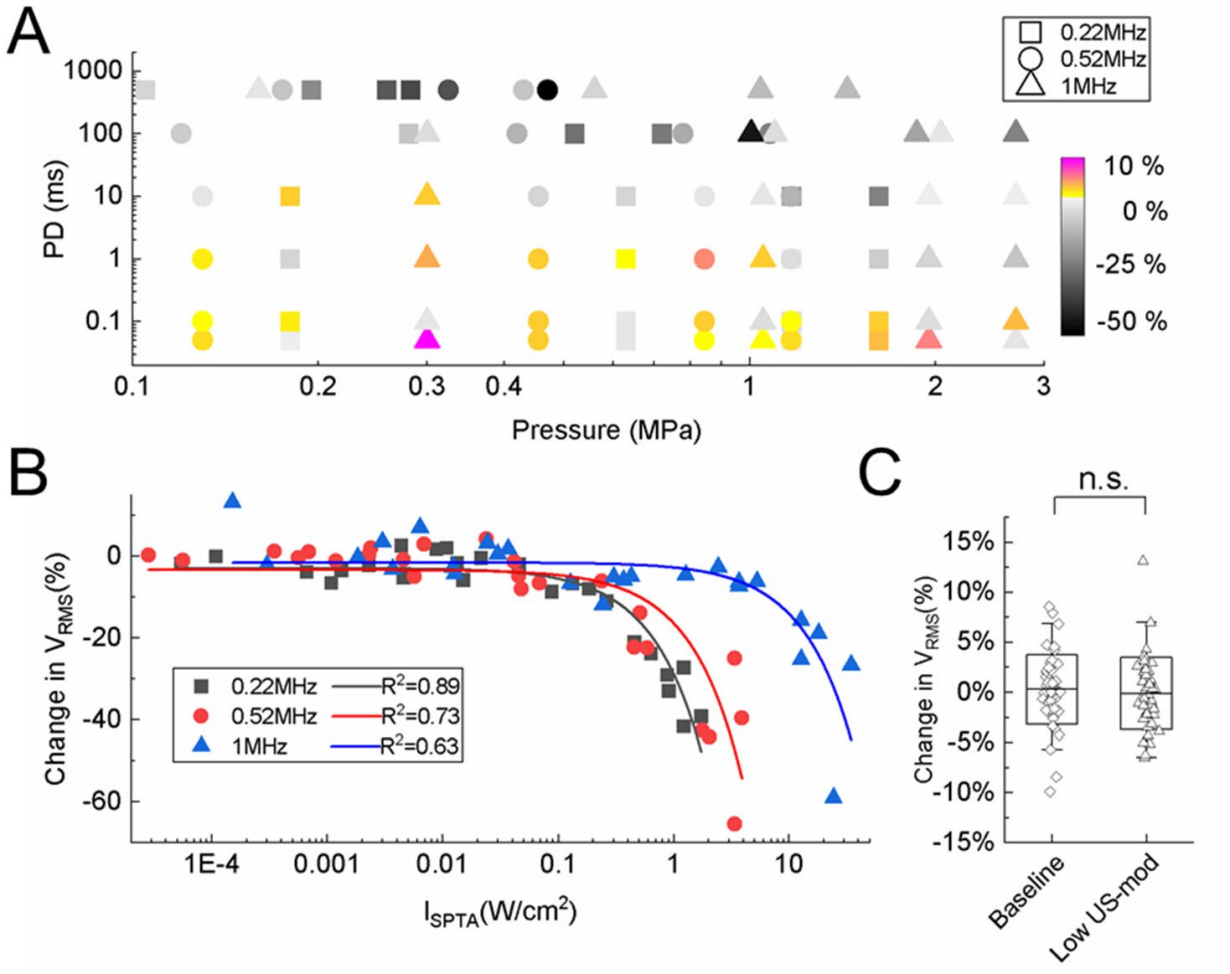
US modulation effects on the sciatic nerve for a wide range of US parameters. **(A)** Parameter response map of pressure and PD space. As PD and pressure increased, US modulation shifted from enhancement or suppression to predominantly suppression effects. **(B)** Change in *V*_RMS_ is plotted as a function of intensity (*I*_SPTA_). Modulation effects shifted from both enhancement and suppression to only suppression as *I*_SPTA_ increased. Lower center frequencies required lower intensities to achieve the same amount of suppression as achieved for higher center frequencies. Based on the curve fits, to elicit a suppression of 50% over 100 trials (1 s TD), it would require 8.33, 11.15, and 32.49 W/cm^2^ with the 0.22, 0.52, and 1 MHz transducer, respectively. Data acquired across five animals. **(C)** Comparison between the change in *V*_RMS_ (mean ± SD) of the baseline paradigm and the US-mod paradigm using low intensities (*I*_SPTA_), which are 0.01, 0.02, and 0.04 W/cm^2^ for the 0.22, 0.52, and 1 MHz transducer, respectively (i.e., portion of the curve where both enhanced and suppressed responses are observed and below intensities where responses become noticeably suppressed). Data were pooled from total sessions (specified as m below) across different animals (specified as n below). The control group (m = 49, n = 16; black) was not significantly different (p = 0.18) from the low US-mod group (m = 41, n = 7; blue), and the distribution of points were similar across groups. The 0.22, 0.52, and 1 MHz transducers were used. n.s., not significant. Data are represented as mean ± SD.

To compare the modulatory effects of different US center frequencies over a wide range of parameters, we performed experiments across five animals with 72 different US parameters using 0.22, 0.52, and 1 MHz transducers. As shown in the parameter response map (Fig. 4A), there were enhancement or suppression effects at low pressures and short PDs, which shifted to predominantly suppression effects at higher pressures and longer PDs. Figure 4B shows the changes in *V*_RMS_ as a function of *I*_SPTA_. Linear regression fits for the changes in *V*_RMS_ as a function of *I*_SPTA,_ where negative indicates suppression and positive indicates enhancement, shows good correlations for the 0.22 (*R*^2^ = 0.89), 0.52 (*R*^2^ = 0.73), and 1 MHz (*R*^2^ = 0.63) transducers. While US at higher intensities (*I*_SPTA_ greater than 0.01, 0.02, and 0.04 W/cm^2^ for 0.22, 0.52, and MHz, respectively) resulted in only suppression cases, US at low intensities showed either suppression or enhancement. However, statistical analysis of changes in *V*_RMS_ showed no significant difference between US-mod paradigms using the low intensities (i.e., *I*_SPTA_ ≤ 0.01, 0.02, and 0.04 W/cm^2^ for 0.22, 0.52, and 1 MHz transducer, respectively) and baseline paradigms across animals (Fig. 4C), demonstrating that the enhancement of nerve activity observed during US stimulation is attributed to the fluctuation of nerve activity over time, and not US. Therefore, these results show that US can significantly suppress but not enhance nerve activity, and greater suppression occurs with larger PD and pressure values (i.e., increases in *I*_SPTA_). Furthermore, at intensities below levels recommended in the FDA guidance for diagnostic ultrasound (e.g., *I*_SPTA_ < 0.72 W/cm^2^ in Fig. 4B for 0.22 and 0.52 MHz transducers), reliable suppression of nerve activity was still possible.

### Control experiments reveal a thermal mechanism for ultrasound suppression of nerve activity

**Figure 5:**
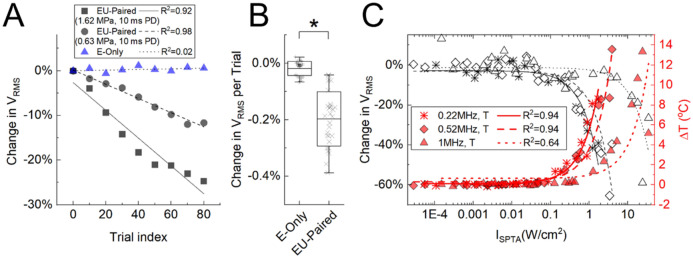
Cumulative suppression of nerve activity over time and increases in temperature during US stimulation. **(A)** Compound action potentials (CAPs) were averaged over a 20-trial window moving forward at a step of 10 trials, spanning a total of 100 trials. Changes in *V*_RMS_ were calculated by comparing the averaged CAP of the moving window with that of the first window and are plotted as a function of the start time of each window in A. Examples for E-only (blue) and EU-paired (1.62 or 0.63 MPa, 10 ms PD, 1 s TD) are plotted. **(B)** Changes in *V*_RMS_ per trial (mean ± SD) for EU-paired groups were significantly lower than E-only groups across 29 sessions in three animals (*p = 5.5E-15, -0.20 ± 0.10% versus -0.01 ± 0.03%). The 0.22 MHz transducer was used in A and B. (**C)** Changes in *V*_RMS_ (black; same data as in Fig. 4B) and temperature (red) are plotted as a function of intensity (*I*_SPTA_) for the 0.22, 0.52, and 1 MHz transducers. Greater nerve suppression was observed at higher *I*_SPTA_, which corresponded to larger increases in nerve temperature. Data are represented as mean ± SD.

To determine if suppression of nerve activity by US occurs immediately or evolves over time during stimulation, we averaged the CAPs across a moving window of 20 trials across the full span of 100 trials, resulting in nine averaged CAPs for an E-Only or EU-Paired data set. The *V*_RMS_ of each averaged CAP was calculated and compared with the *V*_RMS_ of the first averaged CAP. Linear regression fits were performed on the change in *V*_RMS_ values as a function of time (Fig. 5A). For E-Only, the nerve activity did not noticeably change over time. In contrast, EU-Paired exhibited substantial reductions in activity across 100 trials of stimulation, with a *V*_RMS_ per trial (or per second since TD is one second) reduction of 0.31% and 0.16% when US was presented at 1.62 MPa and 0.63 MPa, respectively. Statistical analysis across three animals demonstrates that US significantly suppresses nerve activity over time (EU-Paired versus E-Only paradigm; Fig. 5B). These cumulative effects caused by US suggested that a thermal mechanism may be driving nerve suppression, which was investigated in further experiments described below.

Since the absorption of US will convert energy into heat, which can accumulate in the tissue over time, we monitored the temperature change of the sciatic nerve during US stimulation by placing a hypodermic thermocouple into the agar that was coupled to the transducer and hammocked nerve. In Fig. 5C, we plotted the change in *V*_RMS_ and the change of nerve temperature after 100 trials of US stimulation to assess the relationship between both features. The temperature increased monotonously as *I*_SPTA_ increased during US stimulation. At low intensities, there were minimal temperature changes (< 1 °C), yet reliable suppression of activity was still observed (up to 8.6%, 8.1%, and 11.8% suppression for 0.22, 0.52, and 1 MHz transducers, respectively). As *I*_SPTA_ and temperature increased, substantial suppression of nerve activity was observed, which is consistent with a thermal-dominant mechanism.

**Figure 6:**
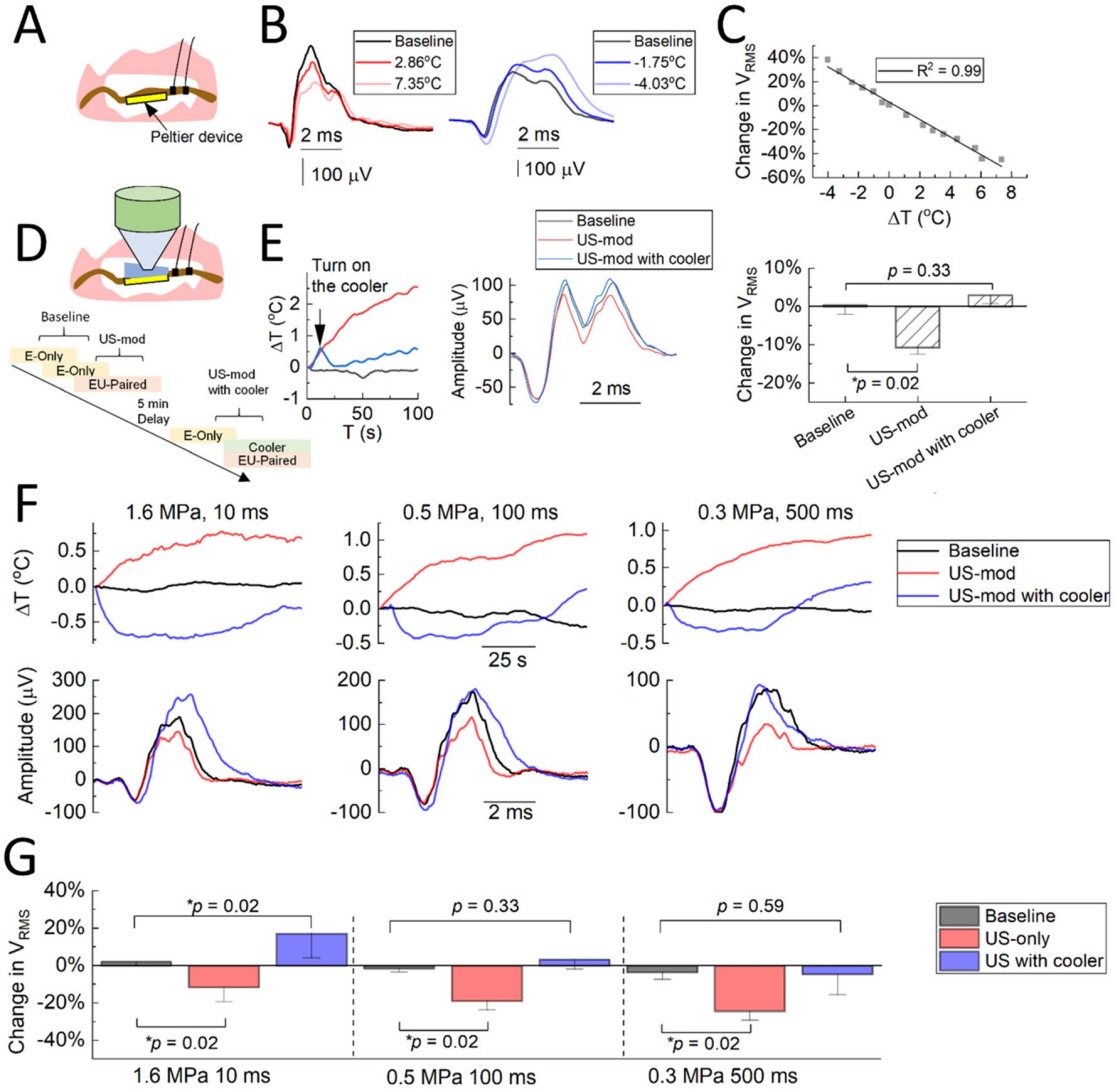
Modulation of nerve activity mediated by changes in nerve temperature. **(A)** Diagram of preparation for heating or cooling the nerve with a Peltier device. Nerve temperature was monitored with a thermocouple. Recording electrodes on the nerve are shown. **(B)** Examples of CAPs at different temperatures relative to baseline CAPs (baseline refers to ES of foot with no US applied). **(C)** Change in V_RMS_ of CAPs is plotted as a function of changes in nerve temperature, revealing a negative correlation. Data from one animal. **(D)** Diagram of preparation for heat dissipation experiments. The cold side of Peltier device was put under the nerve as a cooler to dissipate heat induced by US stimulation of the nerve. Shown in the lower protocol diagram, baseline corresponds to the change in *V*_RMS_ from two consecutive E-Only sessions. US-mod corresponds to change in V_RMS_ between EU-paired session with its preceding E-only session when the cooler was off. US-mod with cooler paradigm corresponds to when the Peltier cooler was on. **(E)** Examples of changes of nerve temperature and nerve activity in one animal. When turning on the cooler (US-mod with cooler), the nerve temperature did not continue to increase as occurs during US stimulation (left plot, red versus blue curves), and suppression of nerve activity was not observed (middle plot). Statistical analysis over three sessions showed that the cooler successfully prevented suppression of nerve activity (right plot). * indicates p = 0.02. **(F-G)** Three diverse US parameters were tested on three animals. Examples of averaged CAPs and temperature changes for different paradigms are plotted in F. US (1.6 MPa, 10 ms PD; 0.5 MPa, 100 ms PD; 0.3 MPa, 500 ms PD) suppressed nerve activity by 11.68 ± 7.77%, 19.06 ± 4.87%, and 24.44 ± 4.74%, respectively. With the cooler, the nerve activity could be enhanced above baseline activity (16.91% ± 12.82% for 1.6 MPa, 10 ms PD) or to baseline activity (0.5 MPa, 100 ms PD and 0.3 MPa, 500 ms). The 0.22 MHz transducer was used in these thermal experiments. Data are represented as mean ± SD.

To verify that a change in nerve temperature results in modulation of nerve activity, we used another thermal source, a Peltier device, to change the nerve temperature by adjusting the polarity and intensity of current delivered to the device (Fig. 6A). By comparing the *V*_RMS_ of CAPs before and during thermal stimulation, we found greater nerve suppression as the nerve temperature increased and greater enhanced activity as the nerve temperature decreased, which can be fitted with linear regression (Fig. 6B, C). Furthermore, we adjusted the nerve temperature towards a constant level during US stimulation by cooling the nerve as US increased the temperature of the nerve (Fig. 6D). As expected, US (1.6 MPa, 10 ms PD, 1 s TD, 0.22 MHz) was unable to noticeably suppress the nerve activity without a steady rise in temperature (Fig. 6E). We tested several US parameters across three animals using the same Peltier device preparation and observed consistent findings in which suppression did not occur or was reversed to enhancement when reducing the nerve temperature (Fig. 6F, G). These findings demonstrate that thermal effects are a dominant mechanism of US suppression of nerve activity.

### Nerve recovery after ultrasound stimulation

**Figure 7:**
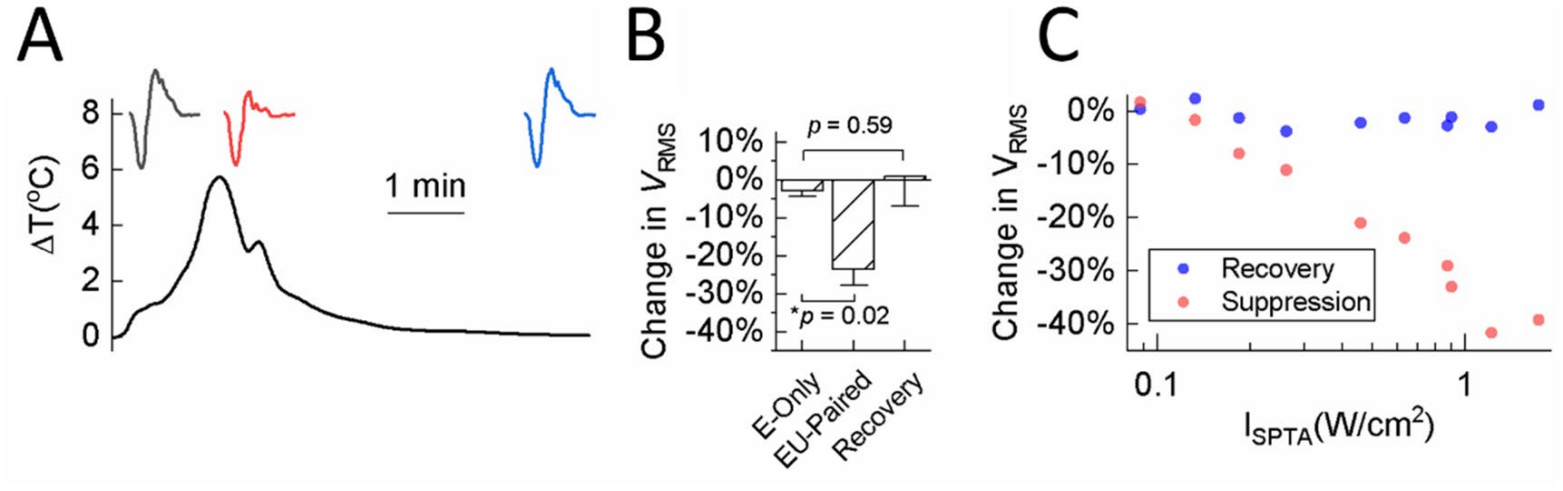
Nerve activity recovery after cessation of US stimulation. **(A)** Averaged nerve activity across 100 trials that was measured multiple times during and after US stimulation are plotted to show full nerve activity recovery over time. During US (0.50 MPa, 100 ms PD, 1 s TD), the CAP (red) was suppressed by 19.17% compared with the baseline CAP before US stimulation (black). Five minutes after cessation of US, the CAP (blue) was fully recovered. (**B)** Changes in *V*_RMS_ (mean ± SD) of baseline, US, and recovery paradigms across three animals. Baseline and US paradigms have been explained in previous figures. The recovery paradigm calculated the change in *V*_RMS_ by comparing the averaged CAPs elicited by E-Only at five minutes after cessation of US with that elicited by E-Only before US stimulation. Error bars represent the standard deviation of *V*_RMS_ across three animals. * indicates US significantly suppressed nerve activity (*p* = 0.02) compared with baseline. p = 0.59 indicates that the nerve activity recovered back to baseline. (**C)** Visualization of the changes in nerve activity (changes in *V*_RMS_) during US (red dots) and after five minutes of recovery (blue dots) for different US stimulation parameters. Data were collected across three animals. All blue dots have returned back to baseline (approximately 0% value; see Supplementary Table S2 showing no significant difference in activity before and after US stimulation), demonstrating that nerve activity can generally recover after cessation of US. The center frequency of the transducer for these data was 0.22 MHz. Data are represented as mean ± SD.

In this study, we did not perform histological assessment of the nerve tissue to determine if US is causing damage to the sciatic nerve. However, we were able to monitor the recovery of nerve activity after US stimulation. As shown in Fig. 7A, the CAPs recovered to baseline activity within five minutes after cessation of US stimulation, which closely aligned with the recovery in nerve temperature back to baseline. Figure 7B presents data from three animals showing that nerve activity is significantly suppressed during US stimulation, which then fully recovers back to baseline within five minutes after cessation of US stimulation. We also tested a diverse set of parameters ranging from 0.046 to 1.728 W/cm^2^ *I*_SPTA_, which included several intensities exceeding levels typically used for diagnostic ultrasound (e.g., FDA guidance level of 0.72 W/cm^2^), and all cases recovered back to baseline (Fig. 7C) with no significant difference in change in *V*_RMS_ during versus after US stimulation (Supplementary Table S2).

## Discussion

### Ultrasound excitation of peripheral nerves

Using pressures up to 5 MPa (*I*_SPTA_ up to 62.48 W/cm^2^ and *I*_SPPA_ up to 833 W/cm^2^), which substantially exceeded levels typically used for diagnostic ultrasound (e.g., FDA guidance of *I*_SPTA_ ≤ 0.72 W/cm^2^ and *I*_SPPA_ ≤ 190 W/cm^2^), we still could not excite the sciatic nerve even when using a wide range of pulse patterns, including different center frequencies, pulse modes, PRFs, and duty cycles tested in our study. Our results are consistent with two studies directly recording nerve activity from completely or partially isolated nerves^17,18^, but are inconsistent with published noninvasive studies reporting US nerve excitation based on indirect measures of nerve activity from non-isolated nerves^11,12^. Based on our observation that low-intensity US can activate the skin and/or surrounding tissue (Fig. 1, Supplementary Fig. S2 and Table S1), one possibility for the discrepancy is that noninvasive US activates other non-nerve tissue (e.g., mechanoreceptors) that leads to skin and muscle activation or body movements, and can be misinterpreted as direct nerve excitation. A previous study has also shown that US can elicit tactile, thermal, and pain sensations^35^, which could lead to a nociceptive withdrawal reflex or unintended body movements. Therefore, future in vivo studies need to separate the targeted nerve from the surrounding tissue if attempting to characterize direct stimulation effects of US on nerve responses. To the best of our knowledge, there are no in vivo studies that have definitively shown that low-intensity US can excite mammalian nerves, which is consistent with the inability to excite nerves across a wide range of US parameters in our study.

Two in vitro studies have shown that US can excite crab leg nerve bundles at 3.5 MPa (562 W/cm^2^ *I*_SPPA_) via an inertial cavitation mechanism^40^, and a US shock wave around 50 MPa can excite a frog sciatic nerve via a cavitation-mediated mechanism^41^. Unlike the unmyelinated crab and frog leg nerves, the guinea pig sciatic nerve has a thick myelin sheath surrounding the nerve fibers and may require even greater pressures to achieve activation through an inertial cavitation mechanism. There are two recent noninvasive studies in mice suggesting that US can excite the mammalian sciatic nerve with very high pressures (e.g., 13 or 28 MPa, greatly exceeding levels recommended in the FDA guidance for diagnostic ultrasound) based on measuring muscle action potentials as a surrogate for nerve activity^15,16^. Therefore, we may not have been able to reach the thresholds of inertial cavitation of the sciatic nerve even at the highest pressure tested in our in vivo preparation, which was limited by the capabilities of our transducers. Future studies will need to test much higher intensity US than evaluated in our study (e.g., ≥ 13 MPa) in an invasive preparation where the nerve is isolated from the surrounding tissue. Extensive histological studies will also be required to demonstrate long-term safety before translating high-intensity as well as low-intensity US stimulation to human applications.

Overall, our study shows that low-intensity US readily activates non-nerve tissue (e.g., skin and/or muscle spindles). However, we did not observe direct excitation of the sciatic nerve with a wide range of US parameters tested in our experiments. The mechanisms underlying US activation of non-nerve tissue and nerves may be different. For example, mechanosensitive ion channels are widely expressed in cortical neurons (e.g., TRPV1, TRPV2, Piezo1, TRPC1, TRPM4, and TRPP1/2 complex), cutaneous mechanoreceptors (e.g., Piezo2, TRPV4), and dorsal root ganglia neurons (e.g., Piezo2)^42–45^ . However, to the best of our knowledge, there is a lack of evidence of the existence of these mechanosensitive ion channels in mammalian axon membranes, such as those in the nerves used in our study. Myelinated nerve fibers mainly express voltage-sensitive sodium and potassium channels, and TREK-1 and TRAAK potassium channels^46–48^, which would induce inhibition but not excitation via mechanical forces of ultrasound^49^. Encouragingly, two other recent studies have shown that targeted US stimulation of the spleen can treat inflammatory arthritis or reduce hyperinflammation, possibly through activation of receptors or cells within end-organs^6,7^. Collectively, with the data presented from our experiments, these findings open up new opportunities for using targeted US to activate receptors in the skin, muscles, and internal organs, rather than nerve bundles, to treat a broad range of health conditions, including pain, inflammation and movement disorders^6,7,50,51^.

### Ultrasound inhibition of peripheral nerves

Based on our findings, US can reliably inhibit nerve activity, in which greater suppression of activity was accompanied by higher *I*_SPTA_ values and increases in nerve temperature. These suppressive effects were eliminated by dissipating the heat generated during US stimulation with a Peltier cooling device coupled to the nerve, indicating that a thermal mechanism is causing suppression of nerve activity. In our study, the nerve temperature was measured with a mini-hypodermic thermocouple, which may induce viscous heating artefact, resulting in overestimating the extent of heating (up to 19% when continuously heating for 5 s)^52^. Therefore, the actual temperature increase accompanying with nerve inhibition may be even lower than we measured, which would be even more encouraging in terms of thermal safety for therapeutic ultrasound. Since we also observed slight suppression of nerve activity when the temperature increased by less than 1 °C, and as a few studies have reported that nerve elongation^53^ or transverse compression^54^ can cause transient changes in membrane ion channels or myelin disruption, there could be a non-thermal contribution to US inhibition of nerve activity. However, our experiments demonstrate that the thermal component of US is the dominant mechanism driving reduction of nerve activity.

Encouragingly, from a clinical perspective, US nerve modulation was reversible. After tens of seconds of US stimulation, nerve activity could be significantly suppressed. These suppressive effects remained for several minutes and then fully recovered back to baseline activity. Other US studies using higher intensity and longer duration stimuli showed that full recovery could take as long as 1–2 hours^32,55^. Future safety studies will need to investigate if different US stimulation parameters, especially higher intensities and longer durations, can reliably inhibit nerve activity without noticeable tissue damage or a decline in long-term nerve function. At least with the diverse set of parameters tested in our study, and specifically for lower center frequencies (i.e., 0.22 and 0.52 MHz) with parameters that were generally within the FDA guidance for diagnostic ultrasound^13,14^, US appears to be safe for suppressing nerve activity. The ability to noninvasively achieve reversible nerve suppression can have significant benefits for treating major health conditions in society^26^. One key opportunity is in the field of neuromodulation for pain treatment, where suppression of nerves or cells is attempted with electrical stimulation using high frequency pulses to put neurons into a refractory state^56–59^. A noninvasive US device could be worn on the body to provide intermittent stimulation of the target nerves or cells, in which residual suppression of activity could enable pain reduction for a sufficient period of time. Another major opportunity is for post-operative surgical pain applications, where patients experience severe pain locally within the surgical area for several days and are prescribed opioids or other pain medications. For example, after oral surgery^60,61^ or neck/thyroid surgery^62^, a patient can experience severe post-operative pain, which has been shown to be reduced with injections of lidocaine or other anesthetics that block local nerve activity. A wearable US device could be used in these patients for a short period of time to reduce local nerve activity and pain symptoms. There are also emerging technologies to develop wearable phased array ultrasound transducers with energy focusing and image-guidance capabilities to enable noninvasive targeting of nerves and cells in the body^63,64^. Therefore, US offers a novel treatment approach for various health conditions that does not require surgery and can potentially reduce our society’s dependence on addictive or unfavorable medications.

## Materials and methods

### Animal preparation

The study was approved by the University of Minnesota Institutional Animal Care and Use Committee (approval number 1512-33257A). Experiments were performed on forty-one guinea pigs (400 ± 50 g, Elm Hill, Chelmsford, MA) in accordance with our approved protocol. Animals were anesthetized with an intramuscular injection of ketamine (40 mg/kg) and xylazine (10 mg/kg), and were given periodic supplements (0.1 ml every 45—60 min) to maintain an areflexive state. Heart rate and blood oxygenation were continuously monitored via a pulse oximeter and body temperature was maintained at 38.0 ± 0.5 ℃ using a heating blanket and rectal thermometer^1,65–67^. The study was carried out in compliance with the ARRIVE guidelines.

### Surgical procedures

All experiments were performed in a sound attenuating, electrically-shielded recording chamber and controlled by a computer interfacing with TDT System 3 hardware (Tucker-Davis Technology) using custom software written in MATLAB (MathWorks). After fixing the anesthetized animal in a stereotaxic frame, subcutaneous lidocaine was administered around the surgical area, and a craniotomy was performed to expose the right SSC. A burr hole was made in the left skull area to place a ground needle electrode for brain recordings.

For noninvasive US stimulation experiments, the leg hair was shaved and a depilatory cream was applied. The US transducer was positioned above the sciatic nerve region and coupled to the leg with ultrasound gel (Fig. 1A). For invasive US stimulation experiments, subcutaneous lidocaine was administered to the leg area and the sciatic nerve was exposed (Fig. 1B). In the “hammocked” setup, the nerve was separated from the surrounding tissue by wrapping parafilm around the nerve to lift it up into the air and affixing the parafilm to the transducer cone. The nerve was positioned at 1 mm below the cone tip, which is at the focus of US. The transducer was then coupled to the nerve with agar, preserving nerve health and isolation (Fig. 1C).

### Neurophysiological recordings in SSC and sciatic nerve

One 32-channel electrode array (NeuroNexus Technologies), which consists of four 5 mm shanks with eight iridium sites linearly spaced 200 µm along each shank, was inserted perpendicular to the surface of the right SSC to a depth of approximately 1.4 mm (Fig. 1D). The electrode array location selected within SSC showed evoked somatosensory activity in response to US stimulation of the left leg (Fig. 1A) and ES of the left foot (Fig. 1E). The electrode was placed to a depth targeting layer IV, which is the main input layer receiving afferent activity from the thalamus and other cortical regions^68^. Neural signals from SSC were sampled at a rate of 25 kHz, and then passed through analog DC-blocking and anti-aliasing filters from 1.6 Hz to 7.5 kHz. Raw data were digitally filtered between 0.3 and 3.0 kHz for spike detection and analysis.

To assess modulatory effects of US stimulation on sciatic nerve activity, we performed an EU-paired paradigm (Fig. 1E). Stainless steel needles were inserted into the left foot to electrically activate the foot receptors projecting through the sciatic nerve, while platinum-iridium wires were used to wrap around the sciatic nerve distal to the spine to directly electrically stimulate the sciatic nerve. Four-site flexible electrode arrays (provided by Dr. Walter Voit’s Lab, UT Dallas) or silver wires were used to wrap around the sciatic nerve proximal to the spine for nerve recording. The reference electrode was the most distal recording site on the nerve and the ground was placed in the neck or brain region. To avoid distortion of nerve activity caused by ringing or smearing of filtered electrical artifacts, unfiltered raw data were used for analysis of nerve activity. During ES of the sciatic nerve, current may spread across the tissue to activate nearby muscles, eliciting compound muscle action potentials (CMAPs). To distinguish between CMAPs and nerve CAPs, we applied a neuromuscular blocker (Succinylcholine, 0.5 mg/kg, IM) over the surrounding muscles^39^, which minimized confounding muscle responses sensed by the sciatic nerve recording sites. As shown in Supplementary Fig. S4, the true nerve CAP in response to foot stimulation remained after application of the neuromuscular blocker. The recorded CAP was also less affected by the electrical artifact elicited by ES of the foot compared to that elicited by direct ES of the sciatic nerve. These findings demonstrate that care must be taken with appropriate controls to avoid recording artificial nerve responses associated with CMAPs and electrical artifacts when performing direct ES of nerves. For all of the presented US modulation experiments, ES of the foot instead of direct ES of the sciatic nerve was used to elicit sciatic nerve activity and minimize distortion of the true CAPs recorded from the nerve.

### Ultrasound stimulation setup and parameters

US transducers with center frequencies of 0.22 (Custom model 071–02-SN01, Sonic Concepts), 0.52 (GS500-D30, The Ultran Group), and 1 MHz (H-218, Sonic Concepts) were used in the experiments. Each transducer was attached to a plastic coupling cone filled with degassed, deionized water with an intended focus at 1 mm below the tip of the plastic cone. The cone was examined to ensure there were no bubbles or air pockets surrounding the transducer. The aperture of 0.22 and 0.52 MHz transducers are flat circular while the 1 MHz transducer has a radius of curvature of 62.3 mm. The diameters of 0.22, 0.52, and 1 MHz transducers are 30, 30, and 31 mm, respectively. The aperture of the plastic coupling cone tip (i.e., aperture) and the cone lengths (i.e., distance from transducer face to cone tip) are 3 mm and 50 mm, 4 mm and 67 mm, and 4 mm and 55 mm, for 0.22, 0.52, and 1 MHz transducers, respectively. The transducer was driven by a 200 W RF amplifier (E&I 2200, Electronics & Innovation Ltd.) via a 6 m 50-Ω extension cable. Additional impedance matching network boxes were used for the 0.22 and 1 MHz transducer. The amplifier was triggered by a waveform generator (33500B series, Keysight Technologies Inc.) and the TDT System 3 hardware using custom software written in MATLAB. Ultrasound outputs were quantified using either the peak negative (rarefactional) pressure amplitude of the sonication pulse (in units of Pa) or by the spatial-peak pulse-average (*I*_SPPA_) averaged over the reciprocal of the PRF known as the spatial-peak temporal-average (I_SPTA,_ W/cm^2^). The two measures are related through the following formula,

1 $${I}_{\mathrm{SPPA}}=\frac{{P}^{2}}{2\rho c}$$

2 $${I}_{\mathrm{SPTA}}={I}_{\mathrm{SPPA}}*\frac{\mathrm{PD}}{(1/\mathrm{PRF})}$$

3 $${I}_{\mathrm{SPTA}}={I}_{\mathrm{SPPA}}*\frac{\mathrm{PD}}{\mathrm{TD}}$$

where *P* is pressure, *ρ* is density of water (1000 kg/m^3^; an approximation for tissue), *c* is speed of sound (1540 m/s; an approximation for tissue), PD is pulse duration in ms, and TD is trial duration in ms. PRF is pulse repetition frequency in kHz. In multiple pulses mode, *I*_SPTA_ is calculated using Eq. 2. In single pulse mode, *I*_SPTA_ is calculated using Eq. 3.

The transducer calibration was performed in a tank filled with deionized water under free field conditions using a high sensitivity hydrophone (HNR 0500, Onda Corp), which is further detailed in previous work^1^. Pressure field profiles (Supplementary Fig. S1) were constructed by plotting negative pressure amplitudes of sonication pulses collected at different spatial locations, which were normalized with respect to the highest spatial peak negative pressure measured at the intended focus of the transducer. These pressure values were used to calculate *I*_SPPA_, *I*_SPTA_ and mechanical index for comparison with recommended levels provided in the FDA guidance for diagnostic ultrasound, in which derated pressure values were not used because of negligible attenuation through agar to the target tissue.

We applied a broad range of US parameters to stimulate the sciatic nerve, including single pulse mode with different PDs and multiple pulses mode with PRFs (details shown in Fig. 2). Those parameters were limited by maximum PDs to avoid potential damage to the transducers. Maximum pressures of US tested were 2, 1.3, and 5 MPa while minimum pressures were 0.05, 0.1, and 0.1 MPa for the 0.22, 0.52, and 1 MHz transducer, respectively. In the single pulse mode, the PDs varied from 0.01 to 500 ms for 0.5, 1 and 3 s TDs. The PDs were halved for 0.5 s TD (Fig. 2A, C). Additionally, duty cycles from 0.99% (1 ms PD, 101 ms TD) to 99.01% (100 ms PD, 101 ms TD) were also tested (Fig. 2B) in the single pulse mode. In multiple pulses mode, PRF varied from 10 Hz to 5 kHz with 50% duty cycle and 1 s TD when US was presented at the highest pressures (Fig. 2D, E).

An EU-paired paradigm was used to examine US modulation effects on the activity elicited in the sciatic nerve via ES of the foot. ES ranged from 1 to 2.82 mA using biphasic, charge-balanced, cathodic-leading pulses (205 µs/phase) for foot stimulation to elicit activity in the sciatic nerve. US of the sciatic nerve consisted of a single pulse with PD of 0.05, 0.1, 1, 10, 100, or 500 ms with a maximum pressure of 1.62, 1.17, and 2.7 MPa and minimum pressure of 0.03, 0.05, and 0.16 MPa for the 0.22, 0.52, and 1 MHz transducer, respectively. The offset of US with the 0.22 and 0.52 MHz transducers occurred 1 ms (for 1, 10, 100, 500 ms PD) or 2 ms (for 0.05, 0.1 ms PD) ahead of the onset of ES. To minimize distortion of the recorded nerve activity caused by electromagnetic artifacts from the 1 MHz transducer during stimulation, the onset of US was 950 ms ahead of the onset of ES for the 1 MHz transducer. There were 100 trials for each US stimulation parameter set unless otherwise stated.

### Thermal experiments

In some control experiments (Fig. 6A, D), we used a Peltier device (TE-8–0.45–1.3, TE Technology Inc.) for increasing or decreasing the nerve temperature. One small piece of electrical tape was used to cover the metal surface of the Peltier device to minimize electrical noise sensed by the nerve recording electrodes. The current delivered to the Peltier device was manually adjusted with a DC power supply (TP3005T, Tekpower). When measuring the nerve temperature, a mini-hypodermic thermocouple (HYP0-33–1-T-G-60-SMP-M, OMEGA Engineering Inc.) was placed into the agar that was coupled to the transducer and hammocked nerve or placed on the Peltier device that was contacting the nerve. The thermocouple was connected with a thermocouple data logger (TC-08, Pico Technology) for saving temperature data for offline analysis.

### Data analysis

To calculate the amount of modulation of nerve activity with US application, we calculated the change in root mean square magnitude (*V*_RMS_) of the peaks of the averaged CAPs (across 100 trials) during ES alone versus that of paired ES and ultrasound. For normalization, the averaged baseline during no stimulation was subtracted from each averaged CAP response^69^. Time ranges of negative and positive peaks were visually defined for each animal and the local minima and local maxima in those time ranges were selected (as shown as purple Xs in Fig. 3B) and used to calculate the root mean square magnitude with the following equation:

4 $${\mathrm{V}}_{\mathrm{RMS}}=\sqrt{\frac{{\sum }_{\mathrm{i}=\mathrm{n}}{({\mathrm{V}}_{\mathrm{s}}-{\mathrm{V}}_{\mathrm{b}})}^{2}}{\mathrm{n}}}$$

where n equals the number of peaks used for the calculation, V_s_ equals the peak amplitudes during stimulation, and V_b_ equals the averaged baseline value calculated from the 15 ms before the ES occurs.

### Statistical analysis

The data are presented as the mean ± standard deviation of the mean. CAPs were calculated by averaging every ten trials over 100 trials of E-only or EU-paired to obtain ten V_RMS_ values of CAPs for each stimulus. A two-tailed unequal variance t-test on ranked data^70^ with p = 0.05 was performed for the ten *V*_RMS_ values between conditions for each stimulus. The same t-test was also used for determining the significance between two groups consisting of mean changes in *V*_RMS_ (averaged across all 100 trials of CAPs) for different paradigms. Bonferroni correction was performed for multiple comparisons and specified in the appropriate figure or table caption.

## Supplementary Information

Supplementary Information.

## Data Availability

The data that support the findings of this study are available from the corresponding authors upon reasonable request.
