# Supplementary material for: Ultrasound does not activate but can inhibit in vivo mammalian nerves across a wide range of parameters
Source: Sci Rep. 2022 Feb 9;12:2182. doi: 10.1038/s41598-022-05226-7 (PMC8828880; doi:10.1038/s41598-022-05226-7)
Supplement: Supplementary file 1 — Supplementary Information. [file 41598_2022_5226_MOESM1_ESM.docx]

**Ultrasound does not activate but can inhibit *in vivo* mammalian nerves across a wide range of parameters**

*Supplementary materials*

**Authors**

Hongsun Guo^1,†,*^, Sarah J. Offutt^2,†^, Mark Hamilton^1^, Yohan Kim^2^, Cory D. Gloeckner^1^, Daniel P. Zachs^1^, Jamu K. Alford^2^, and Hubert H. Lim^1,3,4,#^

**Affiliations**

^1^Department of Biomedical Engineering

University of Minnesota, Minneapolis, Minnesota 55455, USA

^2^ Restorative Therapies Group, Medtronic, Minneapolis, Minnesota 55432, USA

^3^Institute for Translational Neuroscience

University of Minnesota, Minneapolis, Minnesota 55455, USA

^4^Department of Otolaryngology, Head and Neck Surgery

University of Minnesota, Minneapolis, Minnesota 55455, USA

†These authors contributed equally

#Senior author

*Correspondence and requests for materials should be addressed to H.G. (guoxx691@umn.edu) or H.H.L. (email: hlim@umn.edu).


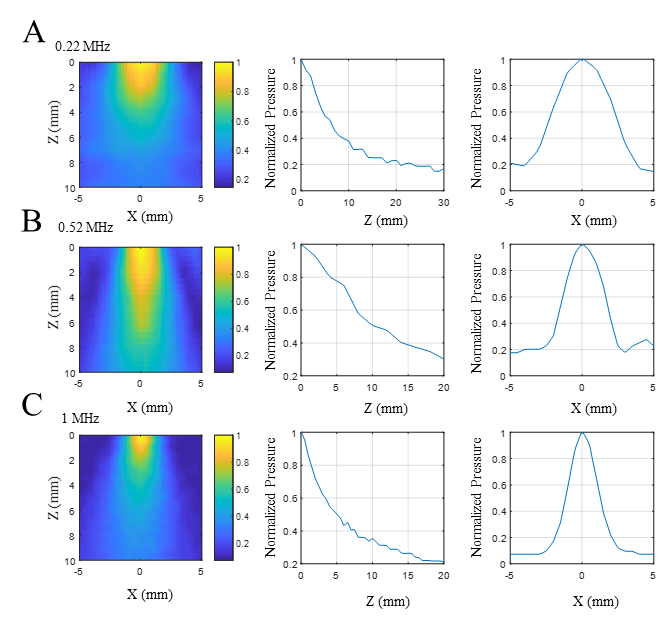


**Fig. S1 Characterization of the 0.22, 0.52, and 1MHz transducers.** The intended focus is at 1 mm below the tip of the plastic cone, where X and Z are all at 0 mm.

**
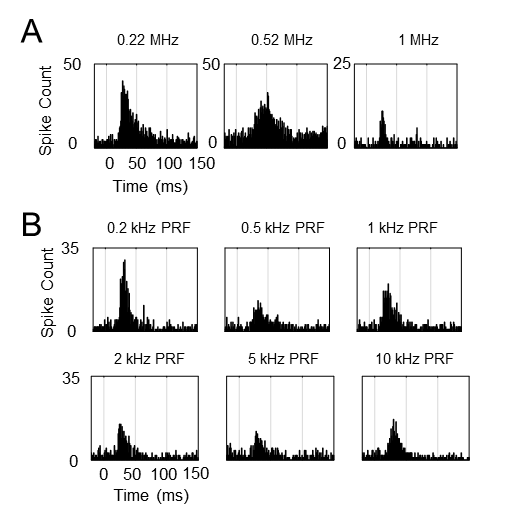
**

**Fig. S2. Examples of neural activity in somatosensory cortex (SSC) in response to noninvasive ultrasound stimulation (US) of the leg surface above the sciatic nerve for different parameters.** Post-stimulus time histograms (PSTHs; 1-ms bins) were plotted across 100 trials. **(A)** Responses to different center frequencies of the US transducers, each with a different pressure: 0.5 MPa for 0.22 MHz, 0.7 MPa for 0.52 MHz, and 2 MPa for 1 MHz. The other parameters were the same for each case (200 Hz pulse repetition frequency [PRF], 2.5 ms pulse duration [PD], 40 pulses, 0.5 s trial duration [TD]). (**B)** Responses to different PRFs but other parameters were the same (100 ms stimulation on time per trial, 50% duty cycle, 0.5 s TD, 0.22 MHz transducer). The onset time of US was at zero for A and B. Additional US parameters that were evaluated that also elicited SSC activity are shown in Supplementary Table S1.

**
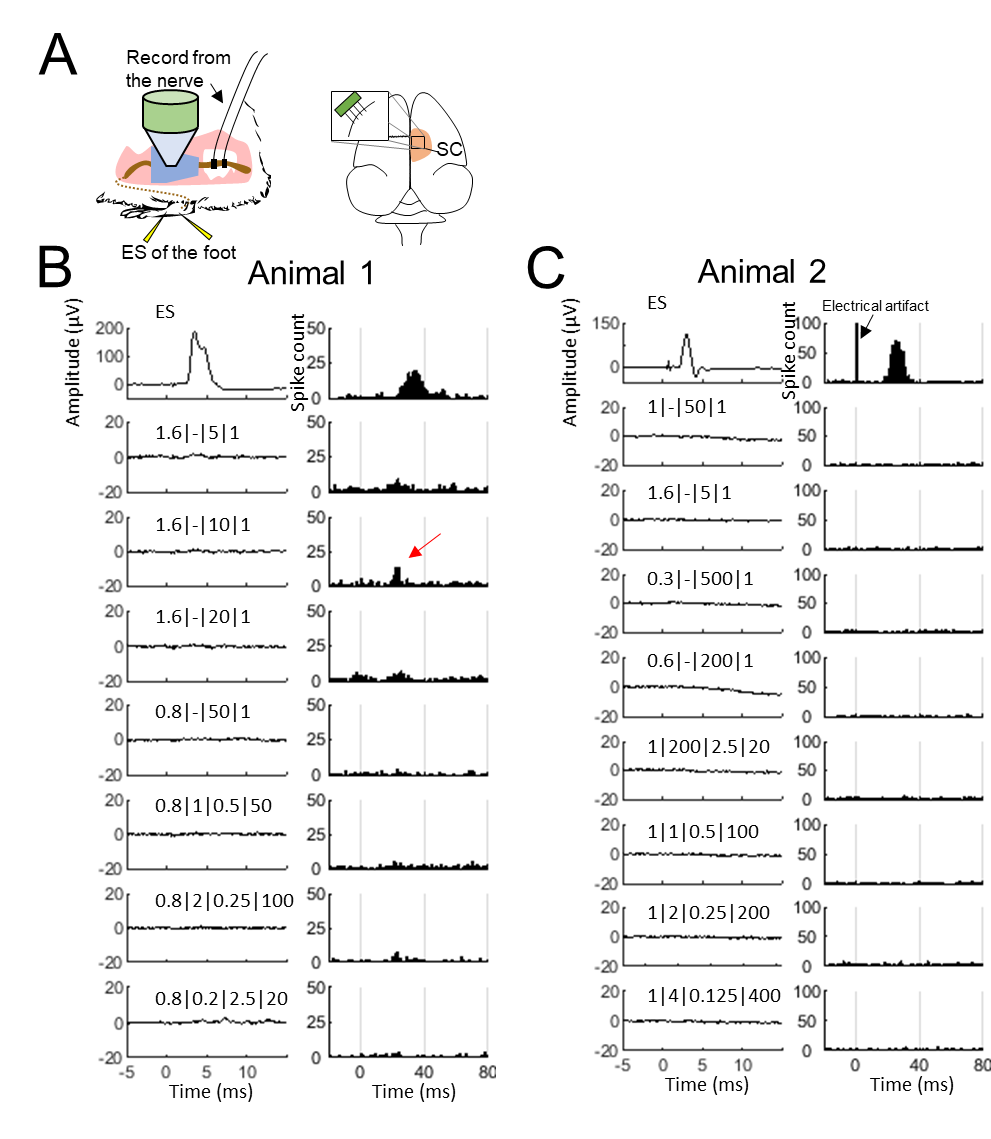
Fig. S3. Examples of neural activity recorded from the sciatic nerve and SSC in response to US stimulation or electrical stimulation (ES) of the sciatic nerve immersed within the muscle cavity.**

**(A)** Illustration shows the preparation of US stimulation of the exposed nerve within the muscle cavity and recording of activity from the nerve and SSC. The transducer was positioned in the muscle cavity and coupled to the distal portion of the nerve with agar. Compound action potentials (CAPs) in response to ES of the foot and US stimulation of the nerve were recorded from the proximal portion of the nerve detached from surrounding tissue [Left]. SSC activity in response to US stimulation of the nerve in the cavity was recorded using a 32-site electrode array (4 shanks, 8 sites per shank) inserted into the SSC [Right].

**(B)** US stimulation of the distal portion of the sciatic nerve did not elicit noticeable CAPs [left plots] but could elicit spike activity in SSC in a few electrode sites in Animal 1 (post-stimulus time histograms, PSTHs, 1-ms bins; right plots). For example, US stimulation (1.6 MPa, single pulse, 10 ms pulse duration [PD]) elicited no CAP response but evoked SSC activity on one site (red arrow) out of 32 sites (other 31 sites not shown for this specific parameter set), suggesting that cortical responses may result from US activation of surrounding tissue and skin receptors due to incomplete isolation of the sciatic nerve. Electrical stimulation (ES, biphasic pulse, 205 µs/phase, level of 2.82 mA) of the foot elicited strong CAPs as well as spike activity across the electrode array in SSC, confirming the nerve was still functional. Only one representative SSC site is shown per parameter set (i.e., per row). US parameters for each row are labeled as: pressure [MPa] | pulse repetition frequency [kHz] or ‘-’ for single pulse | PD [ms] | number of pulses. CAPs were averaged across 100 trials of ES or US stimulation. PSTHs were also binned across 100 trials.

**(C)** Data from another animal. Note that US stimulation did not elicit any CAPs from the sciatic nerve, similar to Animal 1 and consistent with what was observed across a wide range of parameters for the hammocked nerve setup (see Fig. 1, E and H, and Fig. 2). This was the only animal in our study in which US stimulation of the sciatic nerve within the muscle cavity did not elicit any SSC activity, whereas the other animals elicited partial or extensive activity across SSC. To ensure isolation of the sciatic nerve from the surrounding tissue during US stimulation and to minimize confounding activation effects, as occurred in B and in Fig. 1B, a custom “hammocked” preparation was used in this study (see Fig. 1C).


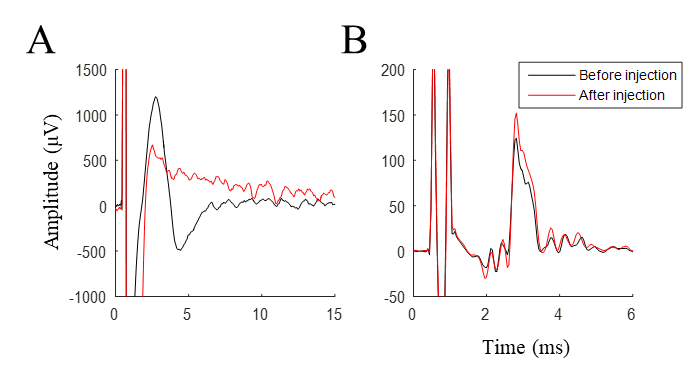


**Fig. S4 Effects of neuromuscular blocker on electrically-evoked nerve activity.** (A) Averaged activity recorded from sciatic nerve in response to 100 trials of biphasic ES (205 µs/phase, 0.1 mA) of the nerve before (black) and after (red) injection of neuromuscular blocker (Succinylcholine, 0.5 mg/kg) to surrounding muscles. (B) Averaged activity recorded from the sciatic nerve in response to 100 trials of biphasic ES (205 µs/phase, 2.82 mA) of the ipsilateral foot before (black) and after (red) injection of neuromuscular blocker to surrounding muscles. Note the large difference in ordinate scale between A and B, in which nerve-based CAPs in B are much smaller in amplitude compared to the muscle activity and confounding electrical artifact that can be recorded on nerve recording electrodes in A.


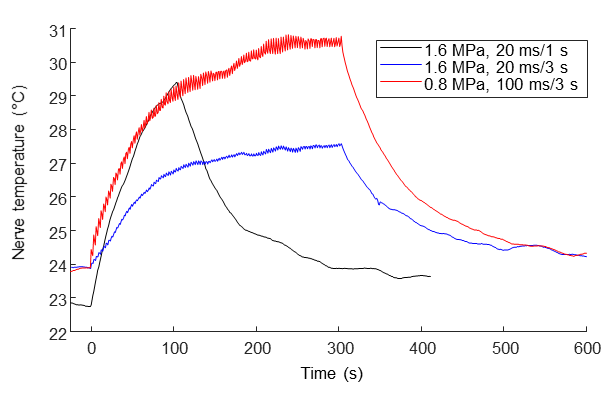


**Fig. S5 Temperature change of the hammocked nerve during US stimulation**. Nerve temperature is plotted over time during 100 trials of US stimulation and for 300 s after cessation of stimulation. Three stimulation conditions using the 0.22 MHz transducer are plotted: *I*_SPTA_ of 1.71 W/cm^2^ (1.6 MPa, 20 ms PD, 1 s TD; black), 0.57 W/cm^2^ (1.6 MPa, 20 ms PD, 3 s TD; blue), and 0.71 W/cm^2^ (0.8 MPa, 100 ms PD, 3 s TD; red). US stimulation started at a time of 0 s on the plot, in which nerve temperature increased during stimulation and then decreased towards baseline once stimulation stopped.

**Table S1 US stimulation parameters activating receptors and/or tissue in the leg based on SSC activity**. Neural activity was recorded in SSC in response to US stimulation. These parameters were tested across three animals. Trial duration was 0.5 s and duty cycle was 50% for cases with multiple pulses presented per trial. For example, when US was presented at 200 Hz PRF, 2.5 ms PD, and 100 ms stimulation on time per trial, the number of pulses was 40. No PRF value is listed for cases with a single pulse per trial. * represents the parameters whose *I*_SPTA_ and *I*_SPPA_ are below FDA safety limits of 0.72 W/cm^2^ and 190 W/cm^2^, respectively.

| **Frequency (MHz)** | **Pressure (MPa)** | **PRF (Hz)** | **PD (ms)** | **Stimulation on time per trial (ms)** |  |
| --- | --- | --- | --- | --- | --- |
| 0.22 | 0.4* | - | 10 | 10 |  |
| 0.22 | 0.4* | - | 50 | 50 |  |
| 0.22 | 0.4 | 200 | 2.5 | 100 |  |
| 0.22 | 0.6* | - | 10 | 10 |  |
| 0.22 | 0.6 | - | 50 | 50 |  |
| 0.22 | 0.6 | 200 | 2.5 | 100 |  |
| 0.22 | 0.8* | - | 10 | 10 |  |
| 0.22 | 0.8 | - | 50 | 50 |  |
| 0.22 | 0.8 | 200 | 2.5 | 100 |  |
| 0.22 | 1 | 200 | 2.5 | 25 |  |
| 0.22 | 1 | 500 | 1 | 25 |  |
| 0.22 | 1 | 1000 | 0.5 | 25 |  |
| 0.22 | 1 | 2000 | 0.25 | 25 |  |
| 0.22 | 1 | 5000 | 0.1 | 25 |  |
| 0.22 | 1 | 10000 | 0.05 | 25 |  |
| 0.52 | 0.8* | - | 1 | 1 |  |
| 0.52 | 0.8* | - | 10 | 10 |  |
| 0.52 | 0.8 | - | 50 | 50 |  |
| 0.52 | 0.8 | 10 | 50 | 100 |  |
| 0.52 | 0.8 | 200 | 2.5 | 100 |  |
| 0.52 | 0.8 | 1000 | 0.5 | 100 |  |
| 1 | 1 | 200 | 0.5 | 20 |  |
| 1 | 2 | 200 | 0.5 | 20 |  |
| 1 | 5 | 200 | 0.5 | 6 |  |
| 1 | 5 | - | 1 | 1 |  |
| 1 | 5 | - | 10 | 10 |  |
| 1 | 5 | - | 30 | 30 |  |

**Table S2 Paradigms used for investigating nerve recovery from US stimulation in one animal.** Each paradigm was performed across 100 trials (1 s TD) and the mean *V*_RMS_ was used to calculate the suppression ratio (fifth column) and recovery ratio (sixth column). Baseline refers to nerve activity before US stimulation. US was applied for 100 trials over 1 minute and 40 s (TD of 1 s) and recovery was assessed at 5 minutes after cessation of stimulation. All other cases had *p* > 0.05. These data support that nerve suppression caused by US can recover after stimulation, at least with the parameters tested in our study. Changes in *V*_RMS_ are also plotted as a function of *I*_SPTA_ in Fig. 6C for better visualization. The *I*_SPPA_ of all paradigms were below FDA safety limit of 190 W/cm^2^. * represents the paradigms whose *I*_SPTA_ exceeds FDA safety limit of 0.72 W/cm^2^.

| **Frequency (MHz)** | **Pressure (MPa)** | **Pulse Duration (ms)** | ***I*_SPTA_ (W/cm^2^)** | $\frac{\boldsymbol{V}_{\mathbf{RMS}}\mathbf{during US}}{\mathbf{Baseline}\boldsymbol{V}_{\mathbf{RMS}}}$  **(%)** | $\frac{\boldsymbol{V}_{\mathbf{RMS}}\mathbf{after recovery}}{\mathbf{Baseline}\boldsymbol{V}_{\mathbf{RMS}}}$  **(%)** | ***p* value** |
| --- | --- | --- | --- | --- | --- | --- |
| 0.22 | 1.62 | 1 | 0.088 | 101.72 | 100.40 | 0.83 |
| 0.22 | 0.63 | 10 | 0.132 | 98.35 | 102.36 | 0.10 |
| 0.22 | 0.105 | 500 | 0.184 | 92.08 | 98.77 | 0.83 |
| 0.22 | 0.28 | 100 | 0.261 | 89.01 | 96.22 | 0.08 |
| 0.22 | 1.17 | 10 | 0.456 | 79.00 | 97.81 | 0.38 |
| 0.22 | 0.195 | 500 | 0.634 | 76.22 | 98.74 | 0.24 |
| 0.22 | 1.62 | 10 | 0.875* | 70.98 | 97.29 | 0.18 |
| 0.22 | 0.52 | 100 | 0.901* | 67.05 | 98.88 | 0.77 |
| 0.22 | 0.27 | 500 | 1.215* | 58.43 | 97.07 | 0.24 |
| 0.22 | 0.72 | 100 | 1.728* | 60.87 | 101.15 | 0.24 |
